# Effets de la Pandémie à Covid-19 sur les Activités Vaccinales D'un Centre de Vaccination de Référence de Treichville en Côte D'ivoire

**DOI:** 10.48327/mtsibulletin.n1.2021.101

**Published:** 2021-04-28

**Authors:** H. Attoh Touré, S. Noufe, K.R. Oussou, K. N'Guessan, S.M. Setchi, A.M.N. Ano, I Tiembre, B.V.J. Bénie

**Affiliations:** 1 Institut national d'hygiène publique, Côte d'Ivoire, BPV 14, Abidjan, Côte d'Ivoire; 2 UFR sciences médicales d'Abidjan, 01 BP 166 Abidjan 01, Côte d'Ivoire

**Keywords:** Covid-19, Vaccination, Observance, Services de vaccination, Institut national d'hygiène publique, Treichville, Abidjan, Côte d'Ivoire, Afrique intertropicale, Covid-19, Immunization, Observance, Immunization services, National institute of public hygiene, Treichville, Abidjan, Ivory Coast, Sub Saharan Africa

## Abstract

**Introduction:**

Depuis le 11 mars 2020, la Côte d'Ivoire est touchée par l'épidémie de coronavirus, déclarée ce même jour comme pandémie par l'OMS. Au 11 mars 2021, soit un an après la pandémie, la Côte d'Ivoire a notifié 36824 cas de patients atteints de Covid-19 et parmi eux 211 sont décédés. Au 31 mai 2020, la Côte d'Ivoire avait déjà notifié 2833 cas et 33 décès. À cette période, de fausses rumeurs circulaient en Afrique sur la mise en place d'essais cliniques sur des candidats vaccins. L'impact de ces rumeurs sur l'utilisation globale des services de santé devait être mesuré et notamment sur les centres de vaccination.

**Objectifs:**

L'objectif de cette étude était de déterminer les effets de la pandémie sur les activités des services de vaccination de l'Institut national d'hygiène publique. L'étude était basée sur les rapports d'activité de son centre de vaccination de Treichville à Abidjan, composé de quatre services: Centre des vaccinations internationales, Service de vaccination des collectivités, Centre antirabique, Unité de vaccination du Programme élargi de vaccination.

**Résultats:**

Au Centre des vaccinations internationales, les activités ont chuté d'environ 50% en mars, de 86% en avril et de 82% en mai par rapport à 2018 et 2019. Pour les vaccinations communautaires, les activités ont diminué d'environ 26% en mars et de 99% en avril et mai. Au Centre de lutte contre la rage, ces diminutions sont estimées à 38% en avril et à 45% en mai. Les réductions les plus importantes concernent les vaccinations contre la fièvre jaune et les méningites.

**Conclusion:**

La baisse de la fréquentation des services de vaccination pourrait augmenter le risque de survenue d'épidémies notamment de fièvre jaune qui sont déjà récurrentes à Abidjan. Des mesures de sensibilisation et de rattrapage vaccinal devraient être entreprises et d'autres études devraient être menées pour déterminer l'impact de la pandémie sur les activités de vaccination.

## Abstract (en)

**Introduction:**

Since March 11, 2020, Côte d'Ivoire has been affected by the coronavirus epidemic, declared that same day as pandemic by WHO. March 11, 2021, one year after the pandemic, Côte d'Ivoire has notified 36,824 cases of Covid-19 patients and among them 211 have died. As of May 31, 2020, Côte d'Ivoire had already notified 2,833 cases and 33 deaths. At that time, false rumors were circulating in Africa about the setting up of clinical trials on candidate vaccines. The impact of these rumors on the overall use of health services had to be measured and in particular on vaccination centers.

**Objectives:**

The objective of this study was to determine the effects of the pandemic on the activities of the immunization services of the National Institute of Public Hygiene in Abidjan, which comprises four departments: International Vaccination Center, Community Vaccination Service, Rabies Center, and Vaccination Unit of the Expanded Program on Immunization. The study was based on activity reports of the immunization services.

**Results:**

At the International Vaccination Center, activities fell by about 50% in March, 86% in April and 82% in May in comparison with 2018 and 2019. Activities of Community Vaccination Service decreased by about 26% in March and 99% in April and May. At the Rabies Control Center, this reduction was estimated at 38% in April and 45% in May. The highest losses were for yellow fever and meningitis vaccines.

**Conclusion:**

The drop in attendance at vaccination services could increase the risk of epidemics, especially yellow fever, which are recurrent in Abidjan. Intensive awareness and catch-up actions should be carried out and further studies performed to assess the impact of the pandemic on immunization activities.

## Introduction

Depuis le 11 mars 2020, la Côte d'Ivoire est touchée par l'épidémie de coronavirus, déclarée ce même jour comme pandémie par l'OMS [3]. Au 11 mars 2021, soit un an après cette déclaration, la Côte d'Ivoire a notifié 36824 cas de patients atteints de Covid-19 et parmi eux 211 sont décédés [2]. Au 31 mai 2020, la Côte d'Ivoire avait déjà notifié 2833 cas et 33 décès [2]. À cette période, de fausses rumeurs circulaient en Afrique sur la mise en place d'essais cliniques sur des candidats vaccins Ces rumeurs colportaient les idées que des pays occidentaux envisageaient de mener sur les populations en Afrique des essais cliniques pour tester l'efficacité de candidats vaccins voire de réaliser des vaccinations sans l'accord des populations notamment chez les enfants [5]. L'impact de ces rumeurs sur l'utilisation globale des services de santé devait être mesuré et notamment sur les centres de vaccination. L'objectif de cette étude était de déterminer les effets de la pandémie à Covid-19 sur les activités des services de vaccination de l'Institut national d'hygiène publique (INHP) à Treichville Abidjan.

## Matériel et Méthodes

Nous avons effectué une étude observationnelle, transversale à visée descriptive au siège de l'INHP à Treichville du 1^er^ mai au 1^er^ juin 2020. L'Institut national d'hygiène publique (INHP) est un établissement public national qui a pour principale mission la prévention et le contrôle des maladies infectieuses transmissibles. Pendant la pandémie, la vaccination à l'INHP a été maintenue aux mêmes horaires il n'y avait pas de confinement du personnel de santé qui y travaillait. L'étude a été menée auprès de ses 4 services de vaccination:

- le Centre de vaccinations internationales (CVI) dédié à la vaccination des voyageurs et de toutes les populations vivant en Côte d'Ivoire non couvertes par le Programme élargi de vaccination (PEV). Il propose des vaccins contre la fièvre jaune, la fièvre typhoïde, la méningite cérébro-spinale ACYW135, le tétanos, les infections à pneumocoque, l'hépatite virale B, la grippe saisonnière, la rougeole, les oreillons et la rubéole
- le Service de vaccination des collectivités (VACCICO) chargé de la vaccination du personnel des entreprises, collectivités et ONG. Ce service propose les mêmes vaccins que ceux du Centre de vaccinations internationales;
- le Centre antirabique (CAR) qui assure uniquement la vaccination des personnes exposées à la rage en post-exposition lorsque le risque est avéré selon les standards de l'OMS ou en pré- exposition
- l'Unité de vaccination du PEV chargée de la protection des enfants de 0 à 11 mois contre les maladies infantiles évitables par la vaccination (tuberculose, poliomyélite, hépatite virale B, diphtérie, tétanos, coqueluche, infections à pneumocoque, infection à *Haemophilus influenzae* de type b, infection à rotavirus, fièvre jaune, méningite à *Neisseria meningitidis* groupe A, rougeole et rubéole), des jeunes filles âgées de 9 ans contre les infections à papillomavirus humain et des femmes enceintes contre le tétanos et la diphtérie.

Pour réaliser cette étude, les rapports de 2018 à 2020 des services sus-cités ont été analysés. Les données ont été collectées dans un fichier Excel. La principale variable d'étude était le nombre de doses de vaccins administrées entre janvier et mai.

Cette étude a été effectuée avec l'autorisation de la direction de l'Institut national d'hygiène publique dans le respect des règles d'éthique.

## Résultats

Par rapport aux mois de mars à mai 2019, les activités vaccinales du CVI ont connu une baisse en 2020 d'environ 50% au mois de mars (6068 doses administrées), 86% en avril (1713 doses administrées) et 82% en mai (2088 doses administrées) (Tableau I). Les baisses les plus importantes ont concerné les vaccins contre la fièvre jaune et les méningites avec une réduction de 90% des doses administrées en avril et en mai (Tableau II). Au niveau de la vaccination des collectivités (VACCICO), les activités ont connu une baisse de 26% en mars (1439 doses administrées), de 99% en avril et mai (moins de 20 doses administrées). Au Centre antirabique, les baisses étaient estimées à 38% en avril et 45% en mai (Tableau I). À l'unité PEV, la baisse d'administration des doses de diphtérie-tétanos-coqueluche-hépatite B- *Haemophilus influenzae* (D-T-C-HépB-Hib), Vaccin polio oral (VPO) et rougeole-rubéole (RR) administrées variait de 10 à 20% (Tableau I).

**Tableau I:** Comparaison des doses totales de vaccins administrées entre janvier et mai de 2018 à 2020 au siège de l'Institut national de santé publique d'Abidjan Comparison of total vaccines doses administered from January to May from 2018 to 2020 at the headquarters of the National Institute of Public Health in Abidjan

	2018	2019	2020	Baisse entre 2019 et 2020 (%)
**Centre de vaccinations internationales**				
janvier	13303	12770	13325	-
février	11994	13218	13989	-
mars	11840	11831	6068	49
avril	11702	12708	1713	86
mai	10761	11546	2088	82
**Service de vaccination des collectivités**				
janvier	1518	587	807	-
février	1613	2339	1096	-
mars	2420	1946	1439	26
avril	3382	2323	17	99
mai	6061	4333	4	99
**Centre antirabique**
janvier	631	537	477	-
février	524	495	490	-
mars	591	517	533	-
avril	527	451	278	38
mai	607	521	285	45

**Tableau II:** Comparaison des doses totales de vaccins contre la fièvre jaune, la méningite, la D-T-C-Hép B-Hib et le RR administrées entre janvier et mai de 2018 à 2020 au siège de l'Institut national de santé publique d'Abidjan Comparison of total vaccines doses of yellow fever, meningitidis, T-D-P-Hep B-Hib and MR administered from January to May from 2018 to 2020 at the headquarters of the National Institute of Public Health in Abidjan

**Fièvre jaune**	**2018**	**2019**	**2020**
janvier	2390	2330	2428
février	2164	2511	2637
mars	2783	2366	1360
avril	2269	2555	144
mai	2136	2488	180
**Méningite AC et ACYW135**	**2018**	**2019**	**2020**
janvier	3751	3354	3459
février	3328	3554	3297
mars	4182	3325	1986
avril	3239	3421	285
mai	3151	3180	458
**D-T-C-HépB-Hib**	**2018**	**2019**	**2020**
janvier	371	388	335
février	491	360	305
mars	412	380	303
avril	359	380	240
mai	171	119	110
**RR**	**2018**	**2019**	**2020**
janvier	235	233	207
février	278	210	219
mars	255	208	230
avril	271	211	131
mai	260	185	134

## Discussion

Contrairement aux autres continents, la progression de la pandémie à Covid-19 en Afrique s'est faite plus lentement. Au 30 mai 2020, alors que le monde comptait 6057853 cas et 371166 décès, le continent africain enregistrait seulement 102133 cas et 2614 décès [4]. Toutefois, les mesures mises en place par les pays, les rumeurs et la crainte du virus ont eu des conséquences sanitaires, sociales et économiques majeures. Au niveau sanitaire, l'une des conséquences est la baisse observée de fréquentation des services de santé, notamment ceux dédiés à la mère et à l'enfant [6].

La baisse du nombre de doses administrées au niveau du CVI pourrait s'expliquer par la fermeture des frontières et l'arrêt des voyages compte tenu de la baisse observée de 90% de la vaccination contre la fièvre jaune.

La fermeture des entreprises, des écoles et l'interdiction des rassemblements de plus de 50 personnes sont des raisons pouvant expliquer la baisse de 99% de la vaccination des collectivités. En effet, la vaccination des collectivités nécessite parfois le regroupement de nombreux bénéficiaires dans des endroits clos.

Seule l'unité PEV a connu une faible baisse d'activité, inférieure à 20%. La sensibilisation individuelle menée par les agents du PEV qui insistaient auprès des mères sur l'importance des 5 contacts pour une vaccination complète des nourrissons peut expliquer cette situation.

La baisse de la fréquentation des services de vaccination pourrait s'expliquer par la crainte de contracter la Covid-19 dans les services de vaccination, la perte des emplois, surtout dans le secteur informel, les difficultés accrues de déplacement des populations, les rumeurs affirmant que des vaccins anti-Covid-19 allaient tuer les populations [5] et la baisse du pouvoir d'achat réduisant l'accès à la vaccination hors PEV. Les activités de communication de masse et digitale n'ont pas été inclusives et suffisamment menées. Les leaders communautaires, religieux, les artistes et les influenceurs n'ont pas été associés à cette communication.

La baisse de la fréquentation des services de vaccination pourrait favoriser le risque de survenue d'épidémies, notamment de fièvre jaune, qui sont déjà récurrentes à Abidjan [1].

D'autres risques sont à craindre notamment la baisse de la protection individuelle des populations contre les maladies courantes à savoir l'hépatite virale B, la fièvre typhoïde, le tétanos et la rage. Concernant la rage, la baisse des activités vaccinales de 50% du Centre antirabique, constitue un risque majeur pour les personnes exposées.

## Conclusion

La pandémie à Covid-19 a eu des effets négatifs sur la fréquentation des services de vaccination en Côte d'Ivoire.

Il est nécessaire de communiquer de manière efficace afin de rassurer les populations et de relancer les activités vaccinales.

Des activités de rattrapage vaccinal doivent également être menées après la pandémie afin d'accroître les couvertures vaccinales.

D'autres études portant sur le système national de vaccination et l'incidence des maladies infectieuses transmissibles devraient être menées pour déterminer l'impact réel de la Covid-19 sur la vaccination dans le pays.

## Conflits D'intérêts

Les auteurs ne déclarent aucun conflit d'intérêt.
